# In the Alphaproteobacterium *Hyphomicrobium denitrificans* SoxR Serves a Sulfane Sulfur-Responsive Repressor of Sulfur Oxidation

**DOI:** 10.3390/antiox12081620

**Published:** 2023-08-16

**Authors:** Jingjing Li, Kaya Törkel, Julian Koch, Tomohisa Sebastian Tanabe, Hsun Yun Hsu, Christiane Dahl

**Affiliations:** Institut für Mikrobiologie & Biotechnologie, Rheinische Friedrich-Wilhelms-Universität Bonn, Meckenheimer Allee 168, 53115 Bonn, Germany; s6jglii2@uni-bonn.de (J.L.); ktoerkel@uni-bonn.de (K.T.); julikoch95@gmx.de (J.K.); s6totana@uni-bonn.de (T.S.T.); s6hshsuu@uni-bonn.de (H.Y.H.)

**Keywords:** *Hyphomicrobium denitrificans*, sulfur oxidation, thiosulfate, SoxR, transcriptional regulation, reactive sulfur species, repressor

## Abstract

In organisms that use reduced sulfur compounds as alternative or additional electron donors to organic compounds, transcriptional regulation of genes for enzymes involved in sulfur oxidation is needed to adjust metabolic flux to environmental conditions. However, little is known about the sensing and response to inorganic sulfur compounds such as thiosulfate in sulfur-oxidizing bacteria. In the Alphaproteobacterium *Hyphomicrobium denitrificans*, one strategy is the use of the ArsR–SmtB-type transcriptional regulator SoxR. We show that this homodimeric repressor senses sulfane sulfur and that it is crucial for the expression not only of *sox* genes encoding the components of a truncated periplasmic thiosulfate-oxidizing enzyme system but also of several other sets of genes for enzymes of sulfur oxidation. DNA binding and transcriptional regulatory activity of SoxR are controlled by polysulfide-dependent cysteine modification. The repressor uses the formation of a sulfur bridge between two conserved cysteines as a trigger to bind and release DNA and can also form a vicinal disulfide bond to orchestrate a response to oxidizing conditions. The importance of the sulfur bridge forming cysteines was confirmed by site-directed mutagenesis, mass spectrometry, and gel shift assays. In vivo, SoxR interacts directly or indirectly with a second closely related repressor, sHdrR.

## 1. Introduction

**Figure 1:**
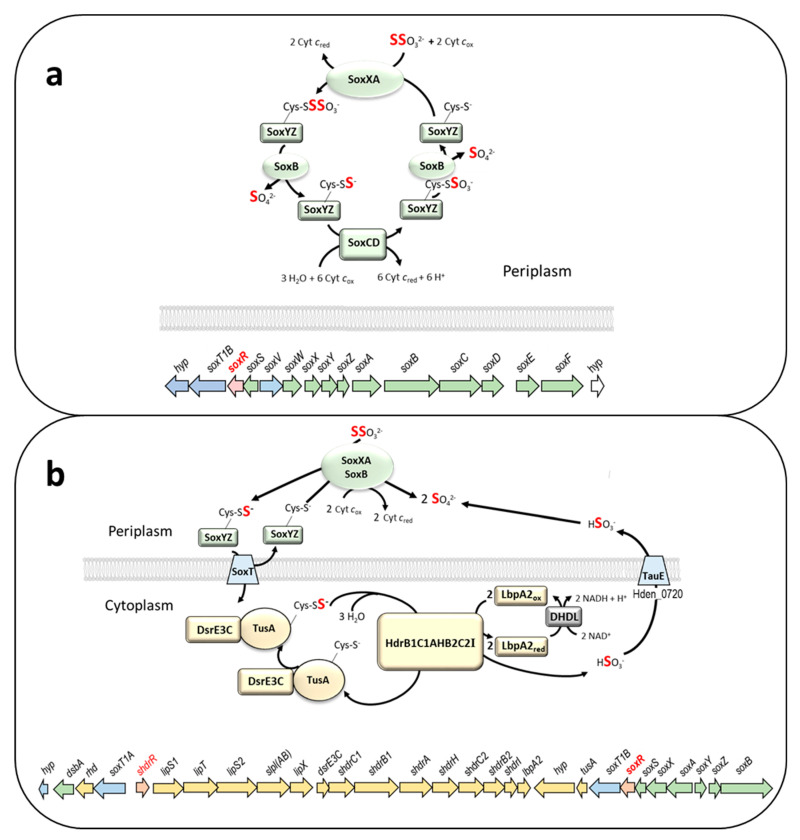
(**a**) Model of the complete periplasmic Sox pathway and exemplary *sox* gene cluster (A6W98_09510 to A6W98_09585) from the Alphaproteobacterium *Rhodovulum sulfidophilum* DSM 1374^T^ (Rhodobacterales, Rhodobacteraceae) [11,12]. SoxS is neither part of the Sox enzyme system nor involved in its regulation [13]. This periplasmic thiol–disulfide oxidoreductase of the Dsb family prevents SoxYZ inactivation by reducing false mixed disulfides [14,15]. (**b**) Model of thiosulfate oxidation and a genome region for sulfur oxidation (Hden_0678 to Hden_0706) in *Hyphomicrobium denitrificans* DSM 1869^T^ (Hyphomicrobiales, Hyphomicrobiaceae) [8]. The *lip* genes encode proteins involved in post-translational assembly of lipoate on the lipoate-binding LbpA2 protein. The truncated Sox system in the periplasm consists of SoxXY, SoxB, and SoxYZ. The sulfane sulfur stemming from thiosulfate and bound to SoxY is transferred to the cytoplasm, possibly via one (or both) of the transporters SoxT1A and Soxt1B, and oxidized to sulfite by the sHdr–LbpA2 system. Sulfite is excreted, probably via TauE, and cannot be effectively oxidized. In panels (**a**,**b**), periplasmic, membrane-bound, and cytoplasmic proteins and the encoding genes are shown in green, blue, and yellow, respectively. Regulator genes are highlighted in red. The *hyp* and *rhd* genes encode a predicted cytochrome P450 and a rhodanese-like protein, respectively. To make them easier to follow, the sulfur atoms that come from thiosulfate are highlighted in bold red.

Thiosulfate (S_2_O_3_^2−^) is a sulfur substrate that is oxidized by the majority of dissimilatory sulfur oxidizers. Its complete oxidation to sulfate is always initiated, and in many cases also completely performed, in the bacterial periplasm and involves the well-studied thiosulfate-oxidizing Sox multienzyme system [1,2,3] (Figure 1a). Three proteins, SoxYZ, SoxXA, and SoxB, are required for the initial steps. The *c*-type cytochrome SoxXA catalyzes the oxidative formation of a disulfide linkage between the sulfane sulfur of thiosulfate and the persulfurated active site cysteine residue of SoxY [4]. Then, SoxB catalyzes the hydrolytic release of the sulfone group as sulfate, leaving the original sulfane sulfur of thiosulfate bound to SoxY [5,6]. The reaction cycle can be fully completed in the periplasm of organisms containing the hemomolybdo-protein SoxCD, which catalyzes the oxidation of SoxY-bound sulfane sulfur to sulfone, followed again by SoxB-catalyzed hydrolytic release of sulfate [7].

Many sulfur oxidizers do not contain SoxCD and have a so-called “truncated” Sox system (Figure 1b) [2]. For complete oxidation to sulfate, truncated Sox systems can be combined with cytoplasmic sulfur oxidation systems. How the sulfur is transferred into the cytoplasm for further oxidation is still a mystery. The Alphaproteobacterium *Hyphomicrobium denitrificans* X^T^ (DSM 1869^T^) is a representative of this group [8] (Figure 1b). In this organism, two genes encoding predicted sulfur compound transporters (SoxT1A and SoxT1B) are located in close proximity to the *sox* genes and the genes for the cytoplasmic sulfane sulfur-oxidizing heterodisulfide reductase-like (sHdr) system (Figure 1b). While the *H. denitrificans* Sox and sHdr proteins have been shown experimentally to be essential for thiosulfate oxidation [8,9,10], evidence for the proposed sulfur transport has not been provided so far.

The obligately heterotrophic *H. denitrificans* oxidizes thiosulfate as an additional electron donor during growth on compounds like methanol [9]. In batch culture, substantial amounts of sulfite are excreted as the product of sHdr-catalyzed sulfur oxidation and accumulate because an enzyme catalyzing efficient sulfite oxidation is not present [9]. Accumulation of sulfite as an intermediate has also been described for some facultatively autotrophic sulfur oxidizing Alphaproteobacteria, e.g., *Rhodovulum* (previously *Rhodobacter*) *sulfidophilum* [16].

(Bi)sulfite (HSO_3_^−^), SO_3_^2−^ is a highly reactive, strong nucleophile and has many toxic effects. Its strong reducing capacity (*E*_0_′ for the sulfate/sulfite couple is −515 mV) contributes to its toxicity and antimicrobial action, which have led to its widespread use as a food preservative [17,18]. Free sulfite can damage DNA through the formation of adducts [19,20,21]. Its toxic effect on mammalian cells has been attributed to the formation of sulfur- and oxygen-based free radicals [22,23] which can in turn react with lipids and proteins [24,25]. The full Sox pathway or the truncated Sox/sHdr combination may be advantageous, despite the intermediate release of sulfite, for organisms such as *H. denitrificans* or *R. sulfidophilum* at low thiosulfate concentrations if removal by other members of the community or chemical oxidation in oxygenated environments keeps sulfite concentrations below inhibitory levels. In any case, the formation of the toxic intermediate sulfite during the oxidation of sulfur compounds, as well as the switching between organic and inorganic electron donors, requires fine-tuning to the environmental conditions.

Accordingly, complex regulatory patterns have been reported for facultative sulfur oxidizers, with upregulation usually occurring only in the presence of metabolizable sulfur substrates, whereas the corresponding genes are thought to always be highly expressed in chemolithoautotrophs restricted to the oxidation of sulfur compounds. In *H. denitrificans* and other Alphaproteobacteria that are not restricted to sulfur oxidation, such as *R. sulfidophilum*, *Paracoccus pantotrophus* or *Pseudaminobacter salicylatoxidans*, the ability to oxidize thiosulfate and, depending on the organism, other reduced inorganic and organic sulfur compounds such as sulfide or dimethyl sulfide, is not constitutive but can be induced by the presence of oxidizable sulfur compounds [9,16,26,27]. While the transcriptional repressor sHdrR is involved in this process in *H. denitrificans* [9], genetic and biochemical studies have identified the related SoxR protein as a major regulator in *P. pantotrophus* and *P. salicylatoxidans* [26,27,28], both of which contain a complete Sox system and are unable to oxidize sulfane sulfur in the cytoplasm.

SoxR is a member of the arsenic repressor (ArsR–SmtB) family of prokaryotic repressors [29,30,31,32]. Members of the ArsR–SmtB family were originally recognized as metal-responsive transcriptional regulators, but there are also members in this family that have been shown to sense reactive oxygen or sulfur species [33]. SqrR from *Rhodobacter capsulatus* and BigR from *Xylella fastidiosa* belong to this group and control the transcription of genes involved in sulfide-dependent photosynthesis and the detoxification of H_2_S derived from associated host plants, respectively [34,35,36]. Knowledge about SoxR is comparatively sparse. While binding regions for the transcriptional repressor have been identified in promoter–operator segments within the *sox* gene clusters of *P. denitrificans* and *P. salicylatoxidans* [26,27], no information is available on factors that control its DNA-binding capacity. It is therefore completely unclear how SoxR senses the presence of oxidizable sulfur compounds and how it then triggers the transcription of sulfur oxidation genes.

Here, we start to close this knowledge gap by first providing information on the general distribution of complete and truncated Sox systems and their co-occurrence with SoxR. Furthermore, we present genetic information for SoxR function in *H. denitrificans*, identify target genes and map its binding sites. The DNA-binding properties of the homodimeric repressor and its response to bridging of the sulfur atoms of two conserved cysteine residues by one to three sulfur atoms are characterized via site-directed mutagenesis, mass spectrometry, MalPEG assays, and electrophoretic mobility shift assays (EMSA).

## 2. Materials and Methods

### 2.1. Bacterial Strains, Plasmids, Primers, and Growth Conditions

Table S1 lists the bacterial strains, primers and plasmids that were used for this study. *Escherichia coli* strains were grown on complex lysogeny broth (LB) medium [37] under aerobic conditions at 37 °C unless otherwise indicated. *Escherichia coli* BL21 (DE3) was used for recombinant protein production. *E. coli* strains 10 beta and DH5α were used for molecular cloning. *H. denitrificans* strains were cultivated in minimal media containing 24.4 mM methanol kept at pH 7.2 with 100 mM 3-(*N*-Morpholino)propanesulfonic acid (MOPS) buffer as described before [8]. Thiosulfate was added as needed. Antibiotics for *E. coli* and *H. denitrificans* were used at the following concentrations (in μg mL^−1^): ampicillin (Ap), 100; kanamycin (Km), 50; streptomycin (Sm), 200; tetracycline (Tc), 15; and chloramphenicol (Cm), 25.

### 2.2. Recombinant DNA Techniques

DNA manipulation and cloning were performed using standard techniques, unless otherwise indicated [38]. Restriction enzymes, T4 ligase and Q5 polymerase were purchased from New England Biolabs (Frankfurt, Germany) and used according to the manufacturer’s instructions. Oligonucleotides for cloning were obtained from Eurofins MWG (Ebersberg, Germany). The GenJET Plasmid Miniprep kit (Thermo Scientific, Waltham, MA, USA) and the First-DNA all-tissue Kit (GEN-IAL GmbH, Troisdorf, Germany) were used for the purification of plasmid DNA from *E. coli* and chromosomal DNA from *H. denitrificans* strains, respectively.

### 2.3. Construction of Plasmid for Deletion of soxR in H. denitrificans

For markerless deletion of the *H. denitrificans soxR* (Hden_0700) gene by splicing overlap extension (SOE) [39], PCR fragments were constructed using the primers P1 fwd up hden_0700, P2 rev up hden_0700, P3 fwd down hden 0700 and P4 rev down hden_0700 (Table S1). The resulting 1.04 kb SOE PCR fragment was cloned into the XbaI and PstI sites of pK18*mobsacB*-Tc [9]. The final construct, pK18*mobsacB*_Tc_Δ*soxR*, was electroporated into *H. denitrificans* Δ*tsdA* and transformants were selected using previously published procedures [8,10]. Single crossover recombinants were Cm^r^ and Tc^r^. Double crossover recombinants were Tc^s^ and survived in the presence of sucrose due to the loss of the vector-encoded tetracyclin resistance and levansucrase (SacB) genes.

### 2.4. Characterization of Phenotypes, Quantification of Sulfur Compounds and Protein Content

Growth experiments with *H. denitrificans* were run in 200 mL medium with 24.4 mM methanol and varying concentrations of thiosulfate in 500 mL Erlenmeyer flasks, as described in [9]. Thiosulfate concentrations, protein content, and specific thiosulfate oxidation rates were determined by previously described methods [9,40]. All growth experiments were repeated three times. Representative experiments with two biological replicates for each strain are shown. All quantifications are based on at least three technical replicates.

### 2.5. RNA Preparation 

Total RNA of *H. denitrificans* was isolated from cells harvested in mid-log phase. *H. denitrificans* strains Δ*tsdA* and Δ*tsdA* ΔsoxR were grown in 50 mL methanol-containing medium at 30 °C with shaking at 250 rpm in 100 mL Erlenmeyer flasks. Cells from 2 mL were harvested by centrifugation at 16,000× *g* for 5 min. The cell pellet was incubated with 500 µL of 10% SDS (sodium dodecyl sulfate) containing 1 mg mL^−1^ lysozyme at room temperature for 5 min. Then 700 µL of TRIzol [41] was added and the mixture was incubated for another 5 min. This step was followed by the addition of 1 mL ROTI^®^Aqua-P/C/I reagent (Carl Roth GmbH, Karlsruhe, Germany), 10 min of incubation and centrifugation at 13,000× *g* for 5 min. RNA purification from the supernatant was achieved with the Monarch Total RNA Miniprep Kit (New England Biolabs, Frankfurt, Germany). gDNA was removed by treating 10 µL samples with an absorption at 260 nm corresponding to ~1 µg RNA with 1 U of RNase-free DNase I (ThermoFisher, Waltham, MA, USA) in the MgCl_2_-containing reaction buffer provided by the manufacturer. RNA concentrations were measured with an NanoDrop Biospectrometer (Eppendorf, Hamburg, Germany). The absence of gDNA was verified using the primers rpoB-denitf and rpoB-denitr [42], which bind only to gDNA and not to the corresponding RNA.

### 2.6. Expression Studies Based on RT-qPCR 

RNA samples of 100 ng were used for RT-qPCR analysis via the Luna Universal One-Step RT-qPCR Kit (New England Biolabs, Frankfurt, Germany) and the CFX Connect^TM^ real-time detection system (Bio-Rad, Munich, Germany) according to the instructions of the manufacturers. The level of *rpoB* mRNA was used as an internal standard [42]. Approximately 200 bp fragments were amplified (see Table S1 in the supplemental material) with an annealing temperature of 60 °C. The RT-qPCR conditions were as follows: 10 min at 55 °C (reverse transcription using random nonamer primers), 1 min at 95 °C (inactivation of the reverse transcriptase and activation of the polymerase), 40 cycles of 15 s at 95 °C, 30 s at 60 °C, followed by melting curve analysis, in which the temperature was increased every 10 s by 1 °C, from a start at 60 °C to 95 °C. Analyses of melting curves and calculations of C_t_ (calculated threshold) values were automatically quantified with the Bio-Rad CFX Manager 3.1 (3.1.1517.0823) software. C_t_ values for each point in time were run in triplicate. Relative expression ratios were calculated by the 2^−ΔΔ*C*T^ method [43].

### 2.7. Cloning, Site-Directed Mutagenesis, Overproduction, and Purification of Recombinant SoxR Proteins

The *soxR* gene was amplified from *H. denitrificans* genomic DNA with primers adding a sequence for an N-terminal Strep-tag and cloned between the NdeI and HindIII sites of pET-22b (+), resulting in pET22b-SoxR-Strep. Cysteine-to-serine exchanges were implemented with the Q5 Site-Directed Mutagenesis Kit (New England Biolabs, Frankfurt, Germany) according to the manufacturer’s instructions and using the primers listed in Table S1. Recombinant SoxR proteins were overproduced in *E. coli* BL21 (DE3) containing plasmids pET22b-SoxR-Strep, pET-22b-SoxR C^50^S, pET-22b-SoxR C^116^S, and pET-22b-SoxR C^50^S C^116^S. The cells were grown in 1 L Erlenmeyer flasks at 37 °C in 400 mL LB medium containing ampicillin up to an OD_600_ of 0.5–0.6. Expression of *soxR* was induced by adding 0.5 mM IPTG (isopropyl-β-d-thiogalactopyranoside). IPTG-induced *E. coli* cells were grown overnight at 20 °C. Cells were harvested at 14,000× *g* for 30 min. Three mL of lysis buffer (100 mM Tris-HCl buffer pH 7.0 and 5 mM EDTA (ethylenediaminetetraacetic acid containing a spatula tip of deoxyribonuclease I and protease inhibitor) were added per g of wet weight for homogenization. Cell lysis was achieved by sonification and followed by centrifugation (16,100× *g*, 30 min, and 4 °C) and ultracentrifugation (145,000× *g*, 1 h, 4 °C). The supernatant was applied to a Strep–tactin affinity chromatography column equilibrated with buffer W (100 mM Tris-HCl, pH 8.0, 150 mM NaCl). The column was washed with six volumes of buffer W and eluted with buffer E (100 mM Tris-HCl, pH 8.0, 150 mM NaCl, 2.5 mM D-desthiobiotin). The protein was assessed for its purity by 12.5% SDS–PAGE (polyacrylamide gel electrophoresis). Pure SoxR proteins were stored on ice in buffer W. Buffer exchange was achieved with Amicon^®^ Ultra-3K centrifugal filters (Merck Millipore, Darmstadt, Germany).

### 2.8. Electrophoretic Mobility Shift Assays (EMSA)

Interactions between proteins and nucleic acids are detected by gel electrophoretic mobility shift assays. In these, solutions of nucleic acid and protein are combined and analyzed for the distribution of nucleic acid species by native polyacrylamide gel electrophoresis. In general, the migration of protein–nucleic acid complexes is slower than that of the corresponding free nucleic acid. The binding reaction mixture (15 μL final volume) contained purified SoxR wild-type or variant protein in various concentrations (up to 700 nM), 2 μL 50% glycerol and 1.5 μL 10 × binding buffer (100 mM Tris-HCl, 500 mM KCl, 10 mM DTT, 5% glycerol, pH 8.0). Reaction mixtures were pre-incubated for 20 min at room temperature, followed by a further 30 min incubation at 30 °C after adding the DNA probe to a final concentration of 17 nM. The DNA probes consisted of a 362-bp fragment covering the entire intergenic region between the *shdrR* (Hden_0682) and the *soxT1A* (Hden_0681) genes, a 180-bp fragment representing the central part of the first product (created with primers EMSA-Fr2-Fr and EMSA_Fr3-Rev), a 177-bp fragment situated between the *shdrR* and the *lipS1* (Hden_0683) genes, a 173-bp fragment situated between the *lipX* (Hden_0687) and *dsrE3C* (Hden_0688) genes, a 176-bp fragment located between the *tusA* (Hden_0698) and *hyp* (Hden_0697) genes, and a 151-bp fragment situated between the *soxA* (Hden_0703) and *soxY* (Hden_0704) genes. All primers used are listed in Table S1. Native 6% polyacrylamide gels were loaded with the reaction mixtures after pre-running the gels at 100 V for 1 h at 4 °C with 0.25 × TBE buffer (25 mM Tris-borate, 0.5 mM EDTA). 0.25 × TBE with 0.5% glycerol was used as the buffer for running the loaded gels for 1 h at 180 V and 4 °C. Gels were subsequently stained for 20 min with SYBR green I. The bands corresponding to SoxR-bound and free DNAs were visualized with a ChemiDoc Imaging System (BioRad, Munich, Germany).

### 2.9. Gel Permeation Chromatography

The size exclusion chromatography column Superdex™ 75 Increase 10/300 GL (Cytiva, Freiburg, Germany) was calibrated using Blue dextran (2000 kDa), conalbumin (75 kDa), bovine serum albumin (67 kDa), ovalbumin (43 kDa), lactoglobulin (35 kDa), carbonic anhydrase (29 kDa), chymotrypsin (23 kDa), and ribonuclease (13.7 kDa). The calibration curve was plotted using the gel-phase distribution coefficient (k_av_) versus the logarithm of molecular weight. K_av_ = (V_e_ − V_0_/V_c_ − V_0_), where V_e_ = elution volume, V_0_ = column void volume (7.94 mL based on Blue dextran elution volume) and V_c_ geometric column volume (24 mL). The column was run in 50 mM Tris-HCl, pH 8.0 and 150 mM NaCl at a flow rate of 0.8 mL min^−1^ using an Äkta FPLC system.

### 2.10. Preparation of Polysulfides

A polysulfide stock solution was prepared according to Ikeda et al. [44] by mixing 1.2 g NaHS × H_2_O and 0.16 g sulfur powder with 3 mL oxygen-free water in a closed 10 mL serum bottle under a nitrogen atmosphere for 1 h at room temperature. Then, the volume was filled up to 10 mL with oxygen-free water. Based on the average length of the resulting polysulfides of four sulfur atoms, their concentration is 0.5 M in the final solution, which can be kept at room temperature for many months. If necessary, the polysulfide solution was diluted with oxygen-free water and immediately used for persulfuration reactions.

### 2.11. Redox Treatments, Persulfuration Reactions, MalPEG Gel-Shift Assays and Mass Spectrometry

A total of 5 µg protein was treated with dithiothreitol (DTT, 1 mM and 5 mM for samples analyzed by mass spectrometry and EMSA, respectively) for reduction, 5 mM CuC1_2_ for oxidation, 0.5 mM polysulfide for persulfuration, 1 mM MalPEG (methoxy-polyethylene glycol maleimide, MW 10,000 g mol^−1^) for PEGylation, or 5 mM iodoacetamide for carbamidomethylation in a final volume of 15 µL containing 100 mM Tris-HCl, pH 8.0 and 150 mM NaCl. When polysulfide, MalPEG, and DTT were applied consecutively, concentrations were 0.5 mM, 5 mM, and 1 mM, respectively. When polysulfide and DTT were applied consecutively, concentrations were 0.5 mM and 10 mM, respectively. Protein samples used in EMSA experiments were reacted with the reagents for 20 min at 25 °C. Samples analyzed by SDS–PAGE were incubated with each reagent for 15 min at 30 °C. Reactions were either stopped by the addition of 5 µL of 4 × non-reducing Roti^®^-Load2 (Carl Roth GmbH, Karlsruhe, Germany) and subjected to 15% SDS–PAGE without boiling the sample or analyzed by mass spectrometry. Samples of 20 µL were desalted by ZiptipC4 Pipette tips (Merck Millipore, Darmstadt, Germany) and measured by matrix-assisted laser desorption/ionization time-of-flight (MALDI-TOF) mass spectrometry at the Core Facility Protein Synthesis & BioAnalytics, Pharmaceutical Institute, University of Bonn.

### 2.12. Distribution of Sox Systems and SoxR: Dataset Generation and Analysis

Archaeal and bacterial genomes were downloaded from the Genome Taxonomy Database (GTDB, release R207). In the GTDB, all genomes are pre-validated, sorted according to validly published taxonomies and are of high quality (completeness minus 5 *contamination must be greater than 50%). One representative of each of the current 65,703 species clusters has been analyzed. The GTDB is based on recently standardized archaeal and bacterial taxonomies derived by normalizing the evolutionary distance between taxonomic levels [45,46]. For bacteria, the database currently lists 148 phyla. For the Archaea, GTDB distinguishes 16 phyla. Open reading frames were determined using Prodigal [47] and subsequently annotated for SoxR, other Sox proteins, and clustering of the respective genes via HMS-S-S with default conditions [48]. Chromatiaceae and Ectothiorhodospiraceae were treated as exceptions as they do not contain contiguous *sox* clusters, but the thiosulfate-oxidizing capabilities and functionality of the Sox proteins have been experimentally established for relevant species [49].

## 3. Results

### 3.1. Distribution of Sox Systems and the SoxR Regulator

**Figure 2:**
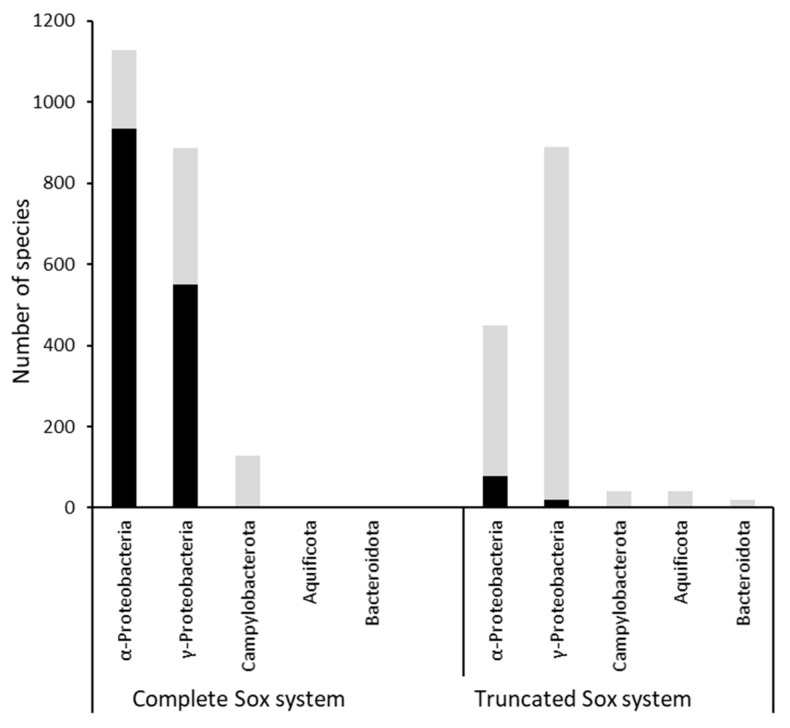
Occurrence of complete and truncated Sox systems among five bacterial phyla. The simultaneous presence of SoxR is indicated in black.

We first asked how complete and truncated Sox systems (Figure 1) are distributed among the prokaryotes and analyzed the genomes available in the Genome Taxonomy Database (GTDB, release R207). In GTDB, all genomes are sorted according to validly published taxonomies. In addition, we asked which groups of these prokaryotes contain a *soxR* that is linked to the other *sox* genes. In order to accurately identify and discriminate between the Sox components, we used HMS-S-S, a tool that specifically finds sulfur metabolism-related proteins [48]. As shown in Figure 2 and Table S2, genes encoding Sox proteins are not found among the Archaea. They exclusively occur in 17 of the currently 169 bacterial phyla distinguished in the GTDB. The highest proportion of species with Sox in a phylum is observed for the Aquificota (54%), followed by the Campylobacterota (30.7%), the Deinococcota (24.3%), and the Proteobacteria (19.3%) (Table S2). The SoxR regulator is strictly confined to the Proteobacteria (Figure 2).

The Aquificota contain exclusively organisms with a truncated Sox system (Table S2), which are strictly chemolithoautotrophic sulfur oxidizers, with a few having additional organoheterotrophic capacity [50]. Among the Sox-containing Campylobacteria, about three quarters rely on a complete system. The type of Sox system varies within a family and even within a single genus. Many Campylobacterota species, e.g., members of the families Sulfurimonadaceae, Sulfurispirillaceae or Sulfurovoraceae, are established chemolithoautotrophic sulfur oxidizers [51,52,53]. In the Deinococcota, the complete Sox system is much more abundant than the truncated version (Table S2), with occurrences in *Thermus* and *Meiothermus* species known as sulfur-oxidizing mixotrophs [54,55]. Among the Bacteroidota, the general abundance of Sox is low, but here we find the obligately photolithoautrophic sulfur oxidizers of the order Chlorobiales [56], all of which encode the truncated set of Sox proteins.

By far the highest absolute numbers of Sox-containing species are found among the Proteobacteria, here exclusively in the classes Alphaproteobacteria and Gammaproteobacteria. The complete Sox system appears more frequently than the truncated version in metabolically versatile members of the alphaproteobacterial families Rhizobiaceae [57] and Rhodobacteraceae [58], while the opposite is true for a number of gammaproteobacterial families, e.g., the Thioglobaceae, Chromatiaceae, Ectothiorhodospiraceae, Thiomicrospiraceae, and Thiotrichaceae (Table S2), all of which contain members with established chemo- or photolithotrophic sulfur-oxidizing capabilities [59,60,61,62,63]. On the other hand, families like the alphaproteobacterial Xanthobacteraceae or the gammaproteobacterial Burkholderiaceae contain species encoding complete or truncated Sox systems in almost equal numbers. The important general rule to emerge from our analysis is that the gene for the SoxR transcriptional regulator is more often linked to the genes for the complete Sox System than to those for the truncated Sox system (Figure 2).

### 3.2. Genetic Evidence for SoxR Function in H. denitrificans

**Figure 3:**
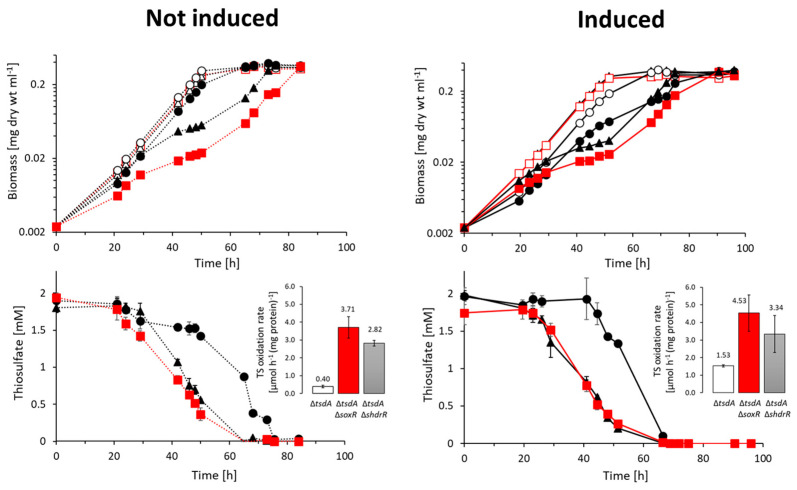
Growth and thiosulfate consumption of *H. denitrificans* Δ*tsdA* (black circles and lines), Δ*tsdA* Δ*shdrR* (black triangles and lines), and Δ*tsdA* Δ*soxR* (red boxes and lines). All strains were grown in medium containing 24.4 mM methanol, either in the absence (open symbols) or in the presence of 2 mM thiosulfate (filled symbols). Pre-cultures contained either no thiosulfate (not induced, broken lines) or 2 mM thiosulfate (induced, solid lines). Thiosulfate concentrations for the different cultures are depicted in the lower panels. Symbol assignments and the color code for specific thiosulfate (TS) oxidation rates are the same as in the upper panels. Error bars indicating SD are too small to be visible for the determination of biomass.

Previously, we showed that the ArsR-type regulator encoded by the first gene of the *H. denitrificans shdr*–*lbpA* operon, sHdrR, functions as a repressor of *shdr* gene expression in the absence of oxidizable sulfur compounds [9]. The phenotypic characterization of a mutant strain lacking the *shdrR* gene indicated an additional regulator involved in the overall process. Indeed, a further candidate transcriptional repressor, SoxR, is encoded downstream of *soxXA* in *H. denitrificans* (Figure 1b). To assign a function for SoxR in transcriptional regulation of the hyphomicrobial *sox* and possibly also the *shdr* and associated genes, we constructed *H. denitrificans* Δ*tsdA* Δ*soxR*, a mutant strain with a markerless deletion of *soxR* in a Δ*tsdA* background. The reference strain *H. denitrificans* Δ*tsdA* lacks thiosulfate dehydrogenase and oxidizes thiosulfate exclusively via the pathway combining Sox and sHdr–LbpA [8,9] (Figure 1b). When grown in the presence of methanol as a carbon source and thiosulfate as an additional electron source, the Δ*tsdA* reference strain excretes sulfite, which causes a growth retardation that is particularly impressive when cultures are inoculated with thiosulfate-induced cells ([9], also compare open and filled circles in the upper right panel of Figure 3). Like the *H. denitrificans* strain lacking the *shdrR* gene, the *soxR*-deficient strain exhibited a high specific thiosulfate oxidation rate and a significantly reduced growth rate even without the induction of pre-cultures (Figure 3). The growth rate increased significantly when thiosulfate was depleted. When pre-cultures were exposed to thiosulfate, both regulator-negative strains exhibited slightly higher specific thiosulfate consumption rates than in the non-induced case, fully in line with the finding that a second regulator is involved in the overall process.

### 3.3. Identification of Genes Controlled by SoxR by RT-qPCR for Different H. denitrificans Strains

**Figure 4:**
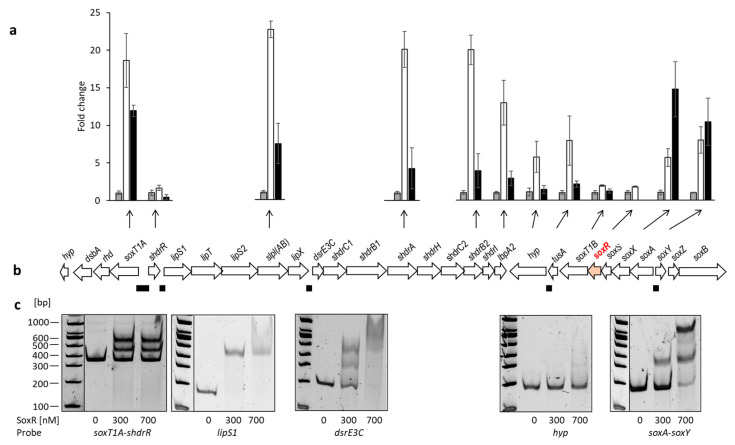
(**a**) Relative mRNA levels of twelve genes located in the *shdr*–*sox* genetic region (depicted in panel; (**b**)) from *H. denitrificans* for the Δ*tsdA* reference strain in the absence (gray columns) and presence of thiosulfate (white columns), as assessed by RT-qPCR. Results for *H. denitrificans* Δ*tsdA* Δ*soxR* are shown by black columns. Results were adjusted using *H. denitrifcans rpoB*, which encodes the β-subunit of RNA polymerase, as an endogenous reference, according to [42]. (**b**) DNA regions tested in EMSA assays for SoxR binding are indicated as black rectangles below the hyphomicrobial *shdr*–*sox* genes. The *soxR* gene is highlighted in red for easier orientation. Fragment sizes: 362 bp for the *soxT1A*–*shdrR* intergenic region, 177 bp and 173 bp for the regions upstream of *lipS1* and *dsrE3C*, respectively. The fragments downstream of *tusA* and between *soxA* and *soxY* had sizes of 176 bp and 151 bp, respectively; (**c**) EMSA analysis of Strep-tagged SoxR with upstream promoter sequence probes of sulfur oxidation-related genes as specified in (**b**). DNA probes of 17 nM were incubated with different amounts of SoxR (300 and 700 nM). Vertical lines separate samples that were run on the same gel but were not directly adjacent.

To examine which genes are affected by the SoxR regulator protein, RT-qPCR experiments were performed, and the transcription levels of twelve genes in the *H. denitrificans* sulfur oxidation genome region were compared in the absence and presence of thiosulfate for the Δ*tsdA* reference strain (Figure 4). In addition, transcription levels were determined for the same genes in the *H. denitrificans* Δ*tsdA* Δ*soxR* mutant in the absence of thiosulfate. All cultures were harvested in the exponential growth phase. The studied genes included *soxT1A*, the first of a set of genes transcribed in the opposite direction of *shdrR*, the gene for the sHdrR regulator, and two of the genes encoding proteins involved in LbpA2 assembly (*lipS1* and *slpl*(*AB*)). LbpA2 is a lipoate-binding protein essential for sulfur oxidation [10]. Four genes were chosen as examples for those encoding the *shdr*–*lbpA2* cytoplasmic sulfane sulfur oxidation system (*dsrE3C*, *shdrA*, *shdrB2*, and *lbpA2*). These genes are followed by genes transcribed in the opposite direction and encoding part of the Sox system (SoxXA), the SoxR regulator, SoxS, which is a periplasmic thiol–disulfide oxidoreductase, as well as a second potential sulfur transporter, SoxT1B, the cytoplasmic sulfurtransferase TusA, and a predicted cytochrome P450 (Figure 1 and Figure 4b). Except for *soxS*, all of these genes were included in the RT-qPCR analysis. Finally, the analysis was extended to *soxY* and *soxB*. These genes follow the previously described genes in the opposite direction in a *soxYZB* arrangement (Figure 1 and Figure 4b).

With the exception of the genes for the two transcriptional regulators, *shdrR* and *soxR*, and *soxT1B*, which is located just downstream of *soxR*, all the genes tested were upregulated at least five-fold when the reference strain was exposed to thiosulfate, with the strongest responses for *lpl(AB)*, *shdrA*, and *shdrB2* (Figure 4a). In the strain lacking SoxR, transcription of various *sox* and *shdr* genes was much higher than in the reference strain, even in the absence of thiosulfate. The lack of *soxR* most strongly affected transcription of *soxXA* and *soxY* but was also evident for *shdr* genes, *soxT1A*, *lipS1*, and *lpl*(*AB*) (Figure 4a). With the exception of the genuine *sox* genes tested, the reference strain showed a stronger response to the presence of thiosulfate than the Δ*tsdA* Δ*soxR* mutant in its absence. This observation clearly points to the presence of at least one further regulatory element, most probably sHdrR [9]. On the other hand, the strong response of numerous genes, in addition to those for the genuine Sox system, shows that their transcription is either directly or indirectly affected by SoxR.

### 3.4. Identification of SoxR Target Sites by EMSA

The finding that SoxR affects transcription of genes outside the *sox* operons was unexpected and afforded a closer analysis. To that end, we inspected intergenic regions within the hyphomicrobial sulfur oxidation region and identified four with conspicuous inverted and direct repeats with the potential to serve as repressor binding sites and used them as probes for EMSA (Figure 4b). A 176-bp fragment located upstream of the hypothetical gene Hden_0697 served as a control (Figure 4b). Indeed, SoxR bound to four of the five tested probes (Figure 4c). Among these is the intergenic region between *soxT1A* and the gene for the SoxR-related repressor sHdrR. This region had already been shown to serve as a binding site for sHdrR [9], further emphasizing the notion that the two repressors work intimately together.

### 3.5. Properties of the SoxR protein

**Figure 5:**
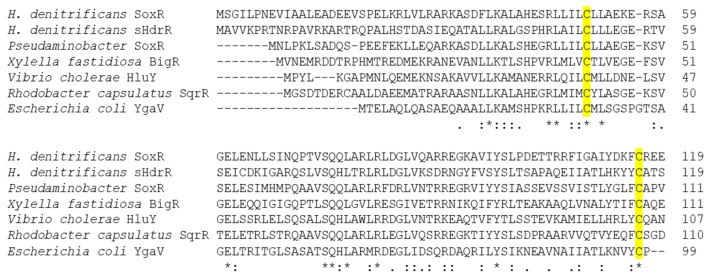
Amino sequence alignment of SoxR homologs. Accession numbers/locus tags and references in the order of appearance: (Hden_0700 [8], Hden_0682 [8,9], WP_010893290 [34], HLYU_VIBCH [65], WP_019171658 [27], ADE85198 [35], and b2667 [66]). An * (asterisk) indicates positions which have a single, fully conserved residue. Conserved cysteine residues are highlighted in yellow. Colons (:) and single dots (.) indicate conserved and semi-conserved amino acids, respectively.

The *H. denitrificans* SoxR protein has a length of 124 amino acids, and a BlastP search (https://blast.ncbi.nlm.nih.gov/Blast.cgi, accessed on 29 June 2023) identified *R. capsulatus* SqrR as the most similar structurally characterized protein. *H. denitrificans* SoxR shows 53%, 52%, 43%, and 42% amino acid identity to *R. capsulatus* SqrR, *P. salicylatoxidans* SoxR, *Xylella fastidiosa* BigR, and *H. denitrificans* sHdrR, respectively. All of these regulators share two conserved cysteine residues, Cys^50^ and Cys^116^, in the hyphomicrobial protein (Figure 5). The equivalent residues in SqrR are required for sensing sulfide [35,64].

**Figure 6:**
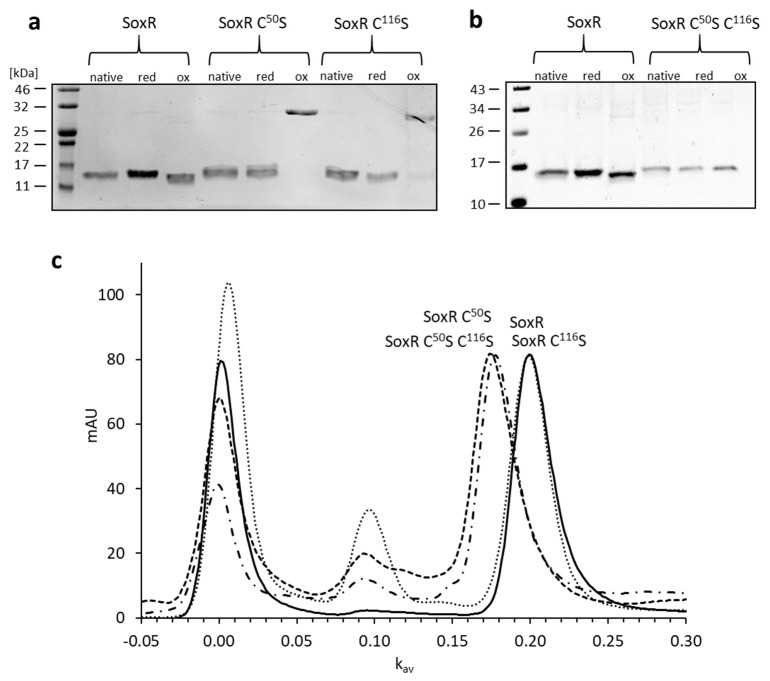
Conformation of SoxR and its variants as analyzed by non-reducing SDS–PAGE analysis (**a**,**b**) and gel permeation chromatography (**c**). For the experiments shown in (**a**) and (**b**), 5 µg of SoxR or its variants were incubated in 15 µL of 100 mM Tris-HCl, pH 8.0 and 150 mM NaCl with either 1 mM DTT or 5 mM Cucl_2_ for 20 min at room temperature, mixed with 5 µL of non-reducing Roti^®^-Load2 (Carl Roth GmbH, Karlsruhe, Germany) and run on 15% SDS polyacrylamide gels. The wild-type SoxR protein is shown twice (**panels a**,**b**) to allow direct comparison with protein variants on different gels. In (**c**), the elution profiles upon gel filtration on Superdex 75 Increase 10/300 are depicted for SoxR, solid line; SoxR Cys^50^Ser, dotted line; SoxR Cys^116^Ser, dashed line; SoxR Cys^50^Ser Cys^116^Ser, dashed-dotted line. SoxR and SoxR Cys^116^Ser dimers elute at a k_av_ of 0.2, corresponding to a molecular mass of 36.7 kDa, whereas SoxR Cys^50^Ser and SoxR Cys^50^Ser Cys^116^Ser elute earlier (k_av_ = 0.174, 41.9 kDa), indicating a more open conformation. The resolution of the column does not allow clear separation of the different tetrameric conformations (k_av_ 0.086 to 0.093, corresponding to 65.9 to 63.6 kDa).

We sought to obtain information about the oligomerization state and conformation of SoxR as well as about the reactivity of the two cysteine residues. To this end, Strep-tagged SoxR as well as variants with serine in place of either one (SoxR Cys^50^Ser and SoxR Cys^116^Ser) or both cysteines (SoxR Cys^50^Ser Cys^116^Ser) were overexpressed in *E. coli*, purified by affinity chromatography, and subjected to reducing and non-reducing SDS–PAGE analysis in the as-isolated state, after reduction with DTT, and after oxidation with CuCl_2_. The same single 15 kDa band was obtained in all cases under reducing conditions (not shown). The band for the oxidized wild-type protein migrated slightly further than those for the as-isolate and reduced proteins under non-reducing conditions (Figure 6a), indicating a more compact structure due to the formation of an intramolecular disulfide bond between Cys^50^ and Cys^116^. The oxidized SoxR Cys^50^Ser and SoxR Cys^116^Ser variants formed intermolecular dimers connected by the remaining cysteine on each of the monomers (Figure 6a). These observations indicated a homodimeric state for the native proteins that allows close contact between the Cys^50^ and Cys^116^ residues, respectively, of the monomers and thus the formation of disulfide bridges under oxidizing conditions.

This conclusion was fully supported by size exclusion chromatography (Figure 6b). All variants, as well as wild-type SoxR, eluted with k_av_ values corresponding to molecular masses between 37.6 and 41.6 kDa, indicating dimerization of the 15.2 kDa monomers. Tetramers were also observed, with the highest abundance for the SoxR Cys^116^Ser variant. All proteins showed a tendency for the formation of higher oligomers in the void volume (Figure 6b). Notably, the Sox Cys^50^Ser single and the Cys^50^Ser Cys^116^Ser variant exchanges led to dimers eluting significantly earlier than those of wild-type SoxR and SoxR Cys^116^Ser, indicating that the loss of Cys^50^ but not that of Cys^116^ leads to a more open, space-demanding conformation of the regulator protein. 

### 3.6. SoxR Binding Properties

**Figure 7:**
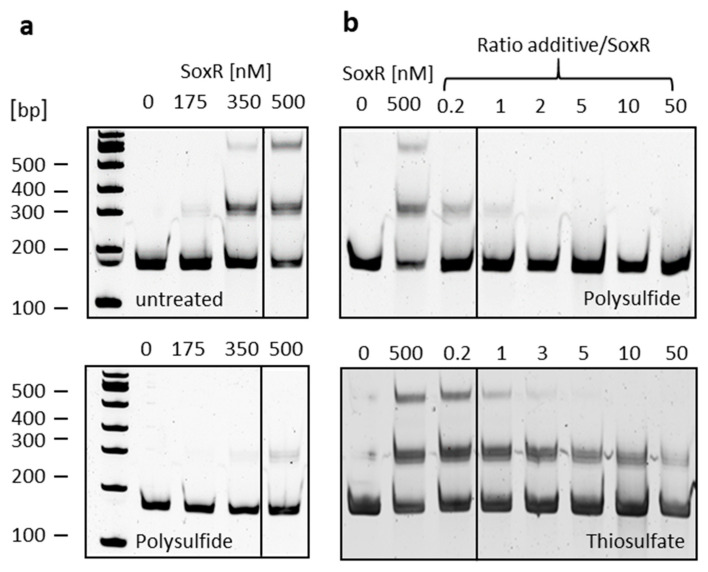
(**a**) EMSA of the 151-bp *soxA*–*soxY* intergenic fragment with increasing amounts of untreated SoxR (**upper panel**) or SoxR pre-incubated with polysulfide in a molar ratio of SoxR/polysulfide of 1:1 (**lower panel**); (**b**) EMSA of the 151-bp *soxA*–*soxY* intergenic fragment with SoxR pre-incubated with increasing amounts of polysulfide (**upper panel**) or thiosulfate (**lower panel**). Vertical lines separate samples that were run on the same gel but were not directly adjacent.

In the next step, EMSA assays were performed that allowed more detailed insights into the binding capacity of SoxR to the intergenic region between the divergently oriented *soxXA* and *soxYZB* genes (Figure 7). SoxR binds to the DNA probe in a concentration-dependent manner and leads to the appearance of two shifted bands indicating two different binding sites (Figure 7a, upper panel). As related proteins respond to persulfuration [35,64], we tested the response of SoxR to treatment with polysulfide, oxidized and reduced glutathione (GSH and GSSG), tetrathionate, sulfite, and thiosulfate in various molar ratios of protein and additive. Whereas GSH, GSSG, tetrathionate, and sulfite had no effect even when present in 50-fold excess compared to the protein (not shown), treatment with polysulfide above a molar ratio of 1 completely prevented binding of SoxR to the target DNA, and a shift was no longer observed (Figure 7a, lower panel, and Figure 7b, upper panel). Thiosulfate also had an effect, albeit a much milder one (Figure 7b, lower panel). The second shifted band disappeared at a ratio thiosulfate/SoxR of 5, and the first band still persisted at a ratio of 50, corresponding to a thiosulfate concentration of 0.2 mM. As outlined in the introduction, the initial steps of thiosulfate degradation occur in the periplasm, and it is therefore unlikely that thiosulfate would ever reach concentrations in the cytoplasm that would be required to elicit a response from SoxR.

**Figure 8:**
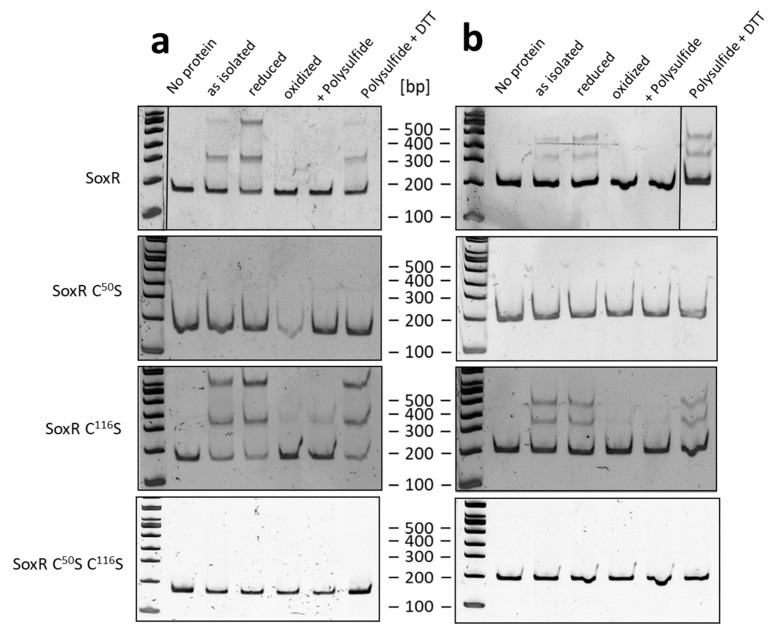
(**a**) EMSA of the 151-bp *soxA*–*soxY* intergenic fragment (17 nM) with 700 nM SoxR wild-type and variant proteins as isolated, reduced with DTT, oxidized with CuCl_2_, treated with polysulfide, and sequentially treated with polysulfide and DTT; (**b**) EMSA of the 180 bp central part of the *soxT1A*–*shdrR* intergenic fragment (17 nM) with 300 nM SoxR wild-type and variant proteins as isolated, reduced with DTT, oxidized with CuCl_2_, treated with polysulfide, and sequentially treated with polysulfide and DTT.

EMSA assays were also performed with the as-isolated, reduced, oxidized, and polysulfide-treated SoxR variants and two different DNA probes (Figure 8). Reduction with DTT led to the same results as those obtained for the untreated proteins, indicating that they are fully reduced upon isolation and remain in this state during storage. Oxidation of wild-type SoxR prevented binding to both tested DNA probes. While the SoxR Cys^50^Ser variant completely lost its DNA binding ability, the Cys^116^Ser variant bound effectively to the DNA probes. When polysulfide-treated wild-type SoxR was reduced with DTT in a second step, the protein regained its DNA-binding capacity, demonstrating that the modification caused by polysulfide was fully reversible by reduction. A response to oxidation or incubation with polysulfide was still observed for the Cys^116^Ser variant, albeit weaker than that of the wild-type protein. This behavior differs significantly from that of the related SqrR from *R. capsulatus*, where variants lacking one of the two conserved cysteines bind to their target DNA but do not show a loss of affinity upon persulfuration [35]. The SoxR variant lacking both cysteines was unable to bind DNA, regardless of the treatments applied.

### 3.7. Redox State and Modification of SoxR

**Figure 9:**
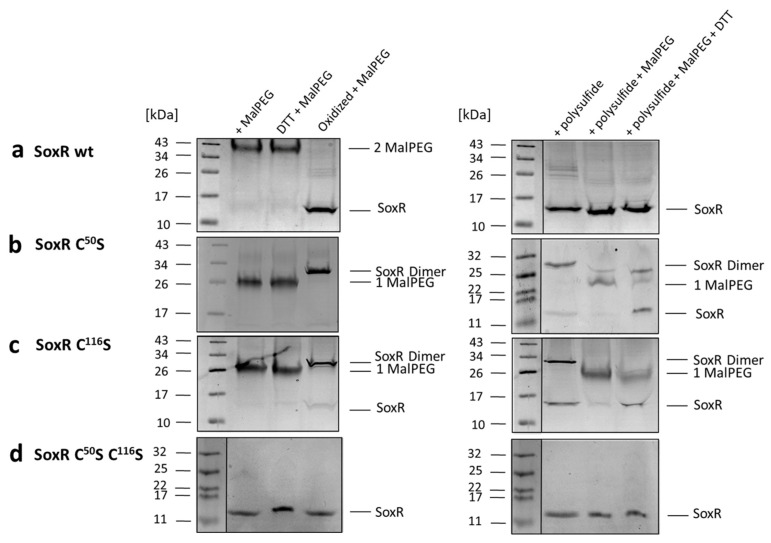
Analysis of *H. denitrificans* SoxR cysteines with MalPEG gel-shift assays in non-reducing SDS–PAGE. Results are shown for the SoxR wild-type (wt) protein (**a**), single (Cys^50^Ser (**b**), and Cys^116^Ser (**c**), and double (Cys^50^Ser Cys^116^Ser (**d**)) variants after MalPEG treatment of the as-isolated, reduced, and oxidized states (**left panels**) as well as after pre-incubation with polysulfide (**right panels**). Polysulfide and MalPEG-reacted samples were further reduced with DTT. Vertical lines separate samples that were run on the same gel but were not directly adjacent.

To clarify the chemical nature of the SoxR modifications by polysulfide and oxidation, gel-shift assays were performed using MalPEG, which selectively labels free thiol groups covalently [67]. The modification can be detected by non-reducing SDS–PAGE since the molecular mass of the protein is increased by ~10 kDa per SH group modified. Treatment of the recombinant wild-type SoxR protein with MalPEG resulted in a single 20 kDa band shift, indicating that it contains two free cysteine residues, as expected (Figure 9a). In contrast, oxidized SoxR did not react with MalPEG (Figure 9a), demonstrating the existence of a disulfide bridge between Cys^50^ and Cys^116^, as also suggested by non-reducing SDS–PAGE in the absence of MalPEG (Figure 6a). MalPEG labeling of the SoxR variants gave the expected results, with the variants carrying one cysteine showing a single 10 kDa shift (Figure 9b,c) and the double mutated variant not reacting with MalPEG as predicted (Figure 9d). After oxidation, the SoxR Cys^50^Ser variant produced exclusively dimers connected by Cys^116^–Cys^116^ disulfide bridges and not reacting with MalPEG (Figure 9b), whereas the Cys^116^Ser variant showed incomplete dimerization. This observation is corroborated by the response of SoxR and its variants to polysulfide (Figure 9, right panels). While the wild-type protein stayed essentially monomeric, i.e., disulfide bonds between protein monomers were not formed, the SoxR Cys^50^Ser variant completely transformed into a dimer stable under denaturing conditions (Figure 9b). The Cys^116^Ser variant behaved differently, with a substantial fraction staying monomeric (Figure 9c). We note that the dimeric fraction of both variants obtained after treatment with polysulfide turned monomeric after incubation with MalPEG, possibly indicating a (poly)sulfur bridge between the remaining cysteine residues that is susceptible to cleavage by the thiol-binding agent. In conclusion, Cys^50^ residues appear less prone to reaction than Cys^116^ residues, just as has been reported for the corresponding cysteines in *R. capsulatus* SqrR [68], and/or they reside further apart from each other in the native SoxR dimer than Cys^116^ residues. 

The next set of reactions was the most revealing. When wild-type polysulfide-treated SoxR was reacted with MalPEG, it behaved just like the oxidized protein, i.e., MalPEG was not bound, indicating the absence of free cysteines (Figure 9a). Instead, one MalPEG was bound to the polysulfide-treated single cysteine replacement variants and could be released upon reduction with DTT (Figure 9b,c). We conclude that in the two latter cases, polysulfide led to the persulfuration of the single remaining cysteines, which then bound MalPEG. In the final step, MalPEG-sulfide conjugates were released by treatment with DTT, and the single cysteine SoxR variants reappeared in their unmodified monomeric form. The situation for wild-type SoxR is completely different. Either polysulfide merely leads to the formation of a Cys^50^–Cys^116^ bridge, or one or more sulfur atoms are enclosed by the two cysteines.

Mass spectrometric analyses finally allowed a clear differentiation between these two possibilities (Table 1, Supplementary Figure S1). For these experiments, MalPEG was replaced by the thiol-modifying agent iodoacetamide, which leads to carbamidomethylation of free Cys sulfhydryl groups and thus adds a mass of 57 Da. As expected, wild-type SoxR gained 57 Da twice after iodoacetamide treatment, whereas the single Cys replacement variants were modified with only one carbamido group. Notably, polysulfide treatment led to persulfuration of all SoxR proteins except for the cysteine-less double replacement variant, which was measured as a control. The SoxR wild-type protein was modified with up to three sulfur atoms (+32 Da each) and did not react with iodoacetamide, demonstrating the formation of an intramolecular tri-, tetra-, or penta-sulfide bond between Cys^50^ and Cys^116^. Although mass spectra do not provide exact quantitative information, peak heights indicate that bridges by two additional sulfur atoms are more abundant than one or three atom bridges for the SoxR wild-type protein, while the majority of the SoxR Cys^50^Ser and Cys^116^Ser variant polypeptides are modified by only one sulfur atom (Supplementary Figure S1).

## 4. Discussion

In this study, we collected a wealth of new information on the transcriptional repressor SoxR. We show that among the more than 70,000 prokaryotic genomes investigated, bonafide *soxR* (i.e., genetically linked to the genes for the SoxYZ sulfur-binding protein and/or catalytic Sox components) occurs exclusively among the bacterial phylum Proteobacteria, where it is more frequently found in gene clusters for complete Sox systems than for truncated Sox systems. Based on the available data, it is difficult to draw general conclusions from this observation. However, it appears that a number of bacteria that operate the truncated Sox system, such as the green and purple sulfur bacteria or members of the Aquificota, are dedicated sulfur-oxidizing chemolithoautrophs without much need for sophisticated transcriptional regulation of the sulfur oxidation machinery.

We show that in the model Alphaproteobacterium *H. denitrificans* SoxR is not only involved in the transcriptional regulation of true *sox* genes but that it also affects the transcription of a number of other genes. In particular, the *shdr* genes, which encode the cytoplasmic sulfur-oxidizing multi-enzyme system required for sulfane sulfur oxidation that cannot be achieved by the truncated hyphomicrobial Sox system, are co-controlled by SoxR. How it interacts with a second, related repressor, sHdrR, that affects the transcription of the same genes [9] is an important research question for the future.

**Figure 10:**
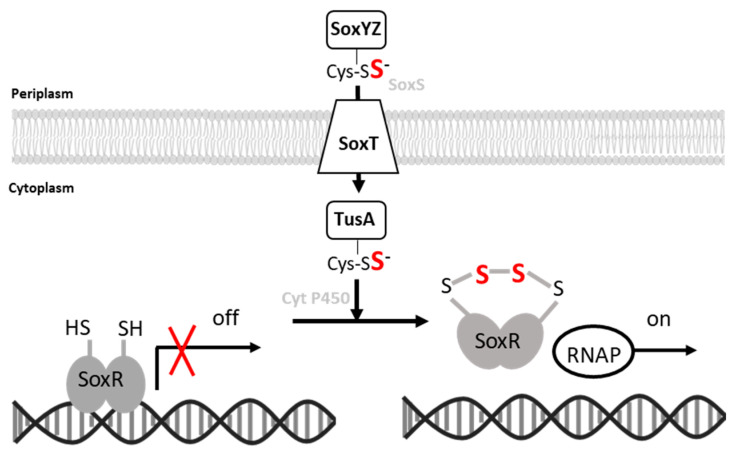
Proposed signal transduction pathway and mode of action of the homodimeric SoxR repressor protein. Established sulfur-binding proteins are printed in black. Sulfane sulfur atoms that come originally from thiosulfate (cf. Figure 1) are highlighted in bold red.

The expression levels of *sox* as well as of *shdr* and associated genes are increased by thiosulfate in wild-type cells and elevated in the *soxR*-deficient *H. denitrificans* mutant, irrespective of the presence of thiosulfate (Figure 4). DNA binding in vitro and probably also transcriptional repression in living cells involve thiol modifications. This can be concluded from the observation that the DNA-binding activity of recombinant SoxR is strongly reduced upon incubation with polysulfide, which leads to persulfuration of the regulator, as proven by reaction with MalPEG (Figure 9) and mass spectrometry (Table 1, Supplementary Figure S1). In polysulfide-treated SoxR, the two conserved cysteine residues can neither be modified by MalPEG nor by iodoacetamide. In addition, polysulfide treatment increases the mass of wild-type SoxR by 32, 64, or 96 Da. These findings can be fully explained by the formation of an intramolecular tri-, tetra-, or penta-sulfide bond formed upon interaction with reactive sulfane sulfur species. Thus, SoxR clearly is not a simple redox sensor switching between dithiol and disulfide states but has been identified as a transcriptional regulator sensing reactive sulfane sulfur species (Figure 10), similar to the related SqrR protein from *R. capsulatus* [35,68]. Notably, the substitution of the two crucial conserved cysteine residues leads to a different outcome for SoxR as compared to SqrR: The lack of Cys^50^ causes complete loss of DNA binding in SoxR, whereas the lack of Cys^116^ creates a variant that tightly binds to its target DNA and is less sensitive to persulfuration than the wild-type protein. In SqrR, both equivalent Cys–Ser variants are DNA-binding competent and do not respond to persulfuration as a signal [35]. Clearly, this difference inspires future research that should also include a detailed inspection of the conformational changes triggered by the formation of a sulfur bridge and resulting in the detachment of SoxR from its target DNA (Figure 10).

The physiological processes involving the various sulfane sulfur-responsive regulators characterized so far [34,35,69,70,71,72] differ fundamentally from those controlled by SoxR. The former mainly regulate stress responses, sense intracellular and extracellular reactive sulfur species, and ensure upregulation of H_2_S oxidation genes for the purpose of detoxification, i.e., they control the removal of excess sulfide and sulfane sulfur, thus contributing to cell survival in the presence of external reactive sulfur species. In contrast, SoxR regulates dissimilatory sulfur metabolism and enables the use of reduced sulfur compounds such as thiosulfate as electron donors for lithotrophic or mixotrophic growth. 

As pointed out earlier, thiosulfate oxidation is initiated in the periplasm, and it is highly unlikely that thiosulfate itself serves as the signaling molecule. Instead, SoxR responds to the presence of low concentrations of sulfane sulfur, which was provided as polysulfide in our in vitro assays. A working hypothesis for how this signal reaches its destination, inspired by the arrangement of the respective genes in *H. denitrificans* (Figure 1b), is presented in Figure 10. It is conceivable that the sulfur bound to the sulfur carrier protein SoxYZ in the periplasm in the course of thiosulfate oxidation reaches the cytoplasm via a YedE-like SoxT transporter [73]. The periplasmic thiol–disulfide oxidoreductase SoxS [15] could be involved in the transfer of the sulfane sulfur to the transporter. Once in the cytoplasm, the sulfur transferase TusA [74] is a possible acceptor protein for the sulfur, which could be passed on from there to SoxR, possibly involving the cytochrome P450 encoded by gene Hden_0697. 

## 5. Conclusions

In conclusion, our study shows that SoxR allows *H. denitrificans* to adapt to changes in thiosulfate availability via thiol persulfidation chemistry and the formation of an intramolecular sulfur bridge, which may involve transporters and sulfurtransferases encoded in the same genetic region. Clearly, much remains to be learned about this regulator, not only in terms of signal transduction but also in terms of crosstalk with its counterpart, sHdrR.

## Figures and Tables

**Table 1:** Mass spectrometry of SoxR and variants after treatment with modifying agents. CAM, carbamidomethylation; S, sulfur. Calculated masses for Strep-tagged SoxR and SoxR Cys^50^Ser, SoxR Cys^116^Ser, and SoxR Cys^50^Ser Cys^116^ without the initiator methionine are 15,212.54 Da, 15,197.54 Da, 15,197.54 Da, and 15,182.54 Da, respectively.

Treatment	SoxR Mass (Da) (Addition: [Da])	SoxR C^50^S Mass (Da) (Addition: [Da])	SoxR C^116^S Mass (Da) (Addition: [Da])	SoxR C^50^S C^116^S Mass (Da) (Addition: [Da])
Native	15,212.8	15,197.3	15,198.2	15,182.3
DTT reduced	15,212.5	15,199.3	15,198.8	nd
CuCl_2_ oxidized	15,210.5	15,196.7	15,196.9	15,182.0
Iodoacetamide	15,328.0 (2 CAM: 2 × 57.07)	15,255.2 (1 CAM: 57.07)	15,255.22 (1 CAM: 57.07)	nd
Polysulfide	15,212.5 15,244.7 (1 S: 32) 15,276.4 (2 S: 64) 15,308.0 (3 S: 96)	15,198.9 15,230.0 (1 S: 32)	15,198.0 15,230.3 (1 S: 32) 15,261.1 (2 S: 64)	15,182.0
Polysulfide + Iodoacetamide	15,212.9 15,244.3 (1 S: 32) 15,275.2 (2 S: 64) 15,306.0 (3 S: 96)	15,198.0 15,286.0 (1 S + 1 CAM: 90)	15,197.6 15,228.5 (1 S: 32) 15,285.4 (1 S + 1 CAM: 90)	15,180.5

## Data Availability

Data are contained within the article and Supplementary Materials.

## Supplementary Materials

The following supporting information can be downloaded at: https://www.mdpi.com/article/10.3390/antiox12081620/s1, Figure S1: Mass spectra for SoxR and variants with Cys-Ser exchanges; Table S1: Strains, plasmids, and primers; Table S2: Distribution of Sox systems and SoxR. References [8,9,42] are cited in Table S1.
