# Supplementary material for: In the Alphaproteobacterium Hyphomicrobium denitrificans SoxR Serves a Sulfane Sulfur-Responsive Repressor of Sulfur Oxidation
Source: Antioxidants (Basel). 2023 Aug 16;12(8):1620. doi: 10.3390/antiox12081620 (PMC10451225; doi:10.3390/antiox12081620)
Supplement: Supplementary file 1 [file antioxidants-12-01620-s001.zip › Figure S1 Mass spectra for SoxR and variants with Cys-Ser exchanges.pdf]

Figure S1. Mass spectra for SoxR and variants with Cys-Ser exchanges

SoxR wildtype

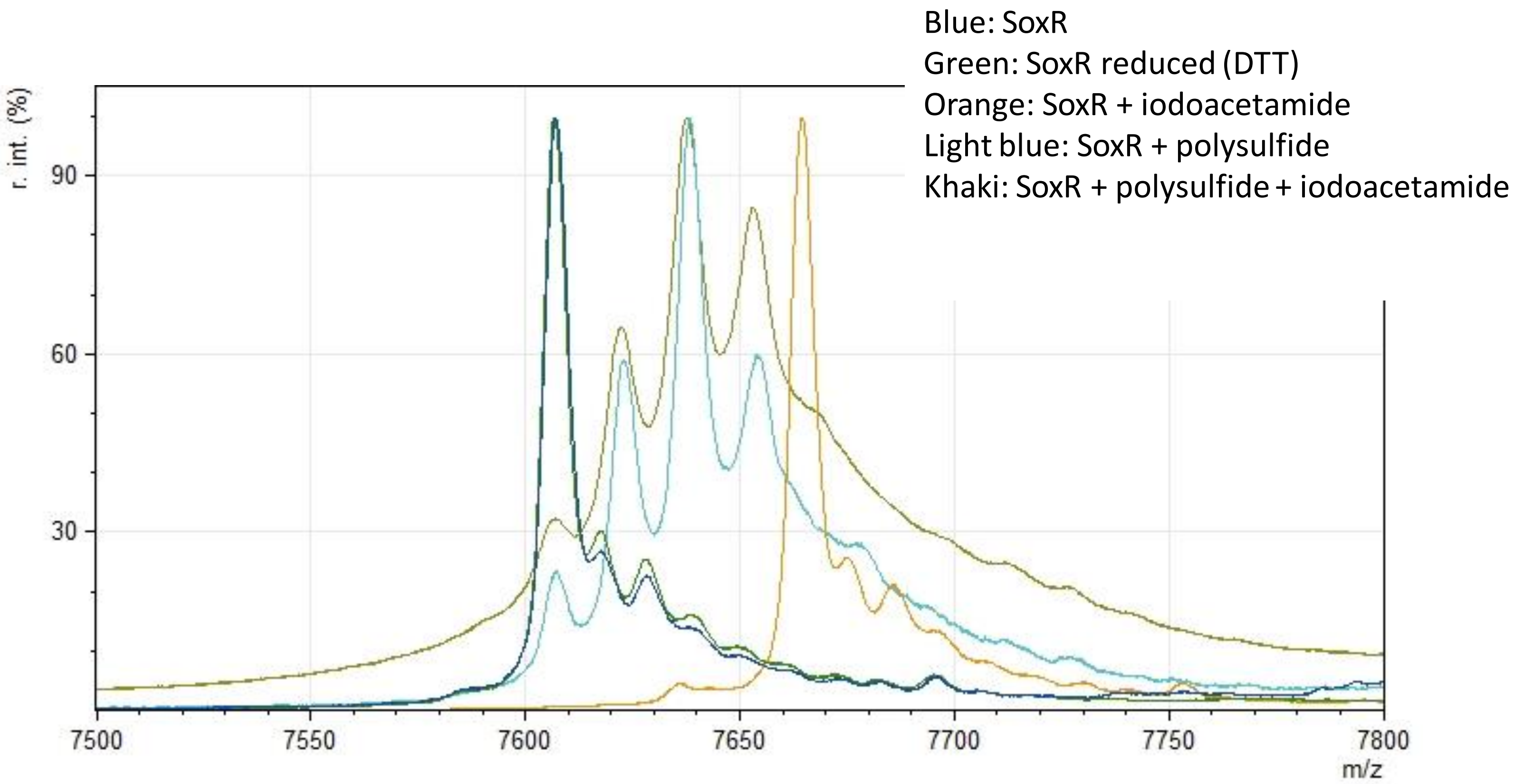

Masses are shown for the *mz*/2 species. Mass spectra obtained for the $CuCl_2$ oxidized protein were almost identical to those for the as isolated protein and are not shown for clarity.

SoxR Cys^{50}Ser

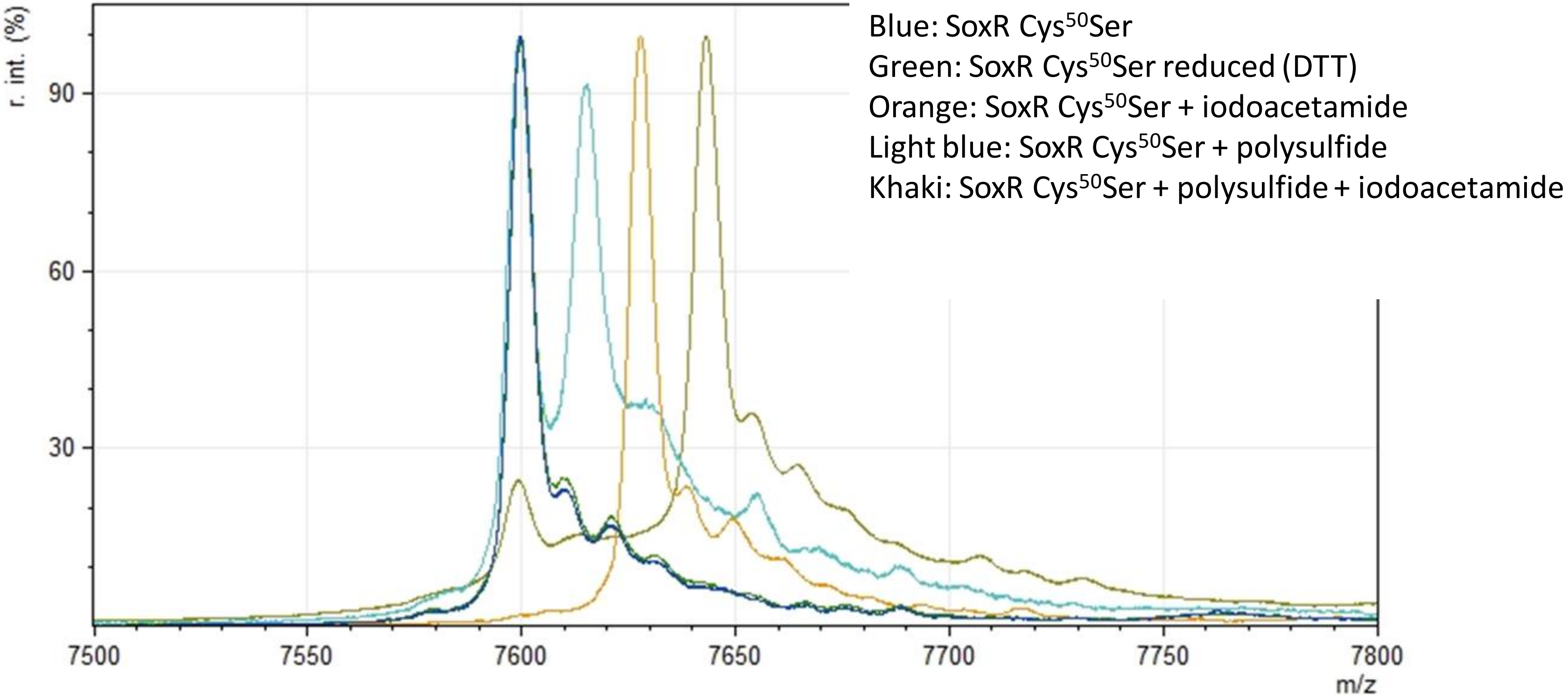

Masses are shown for the *mz*/2 species. Mass spectra obtained for the $CuCl_2$ oxidized protein were almost identical to those for the as isolated protein and are not shown for clarity.

SoxR Cys^{116}Ser

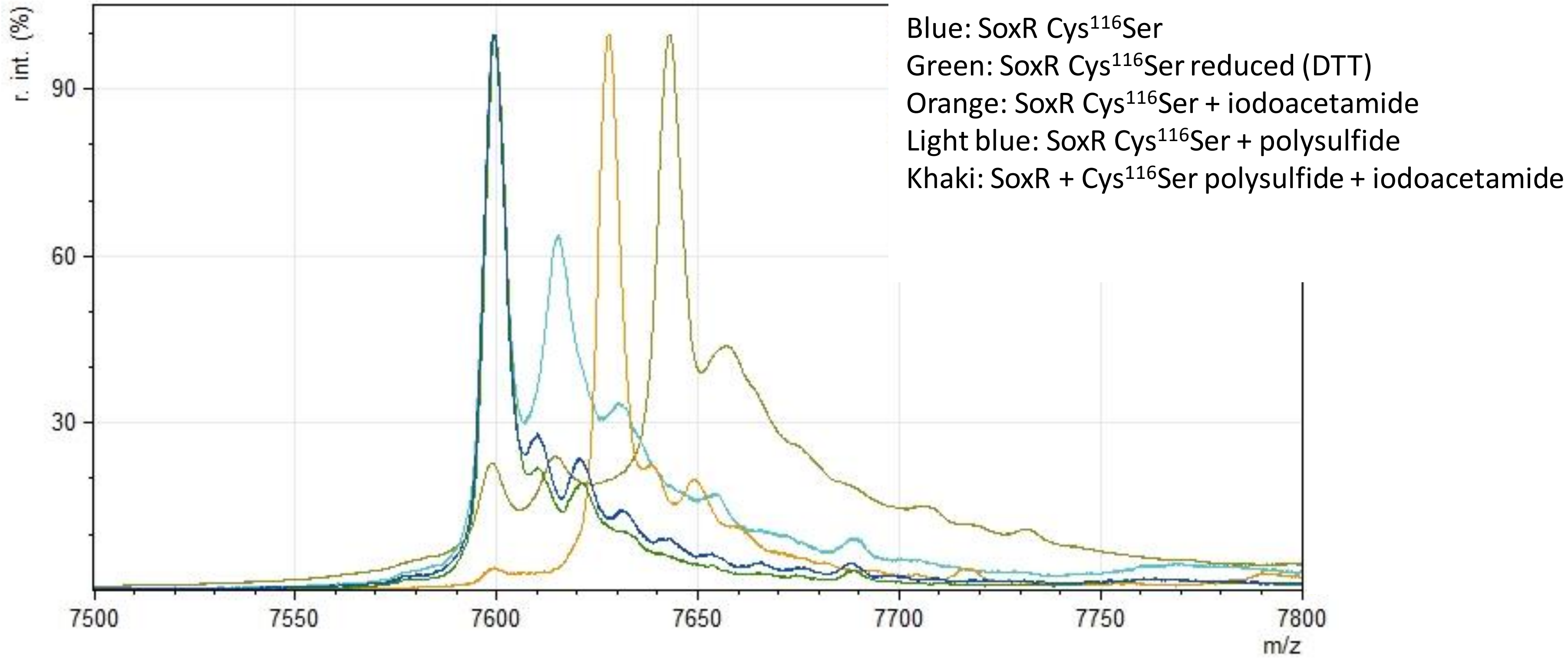

Masses are shown for the *mz*/2 species. Mass spectra obtained for the $CuCl_2$ oxidized protein were almost identical to those for the as isolated protein and are not shown for clarity.

SoxR Cys^{50}Ser Cys^{116}Ser

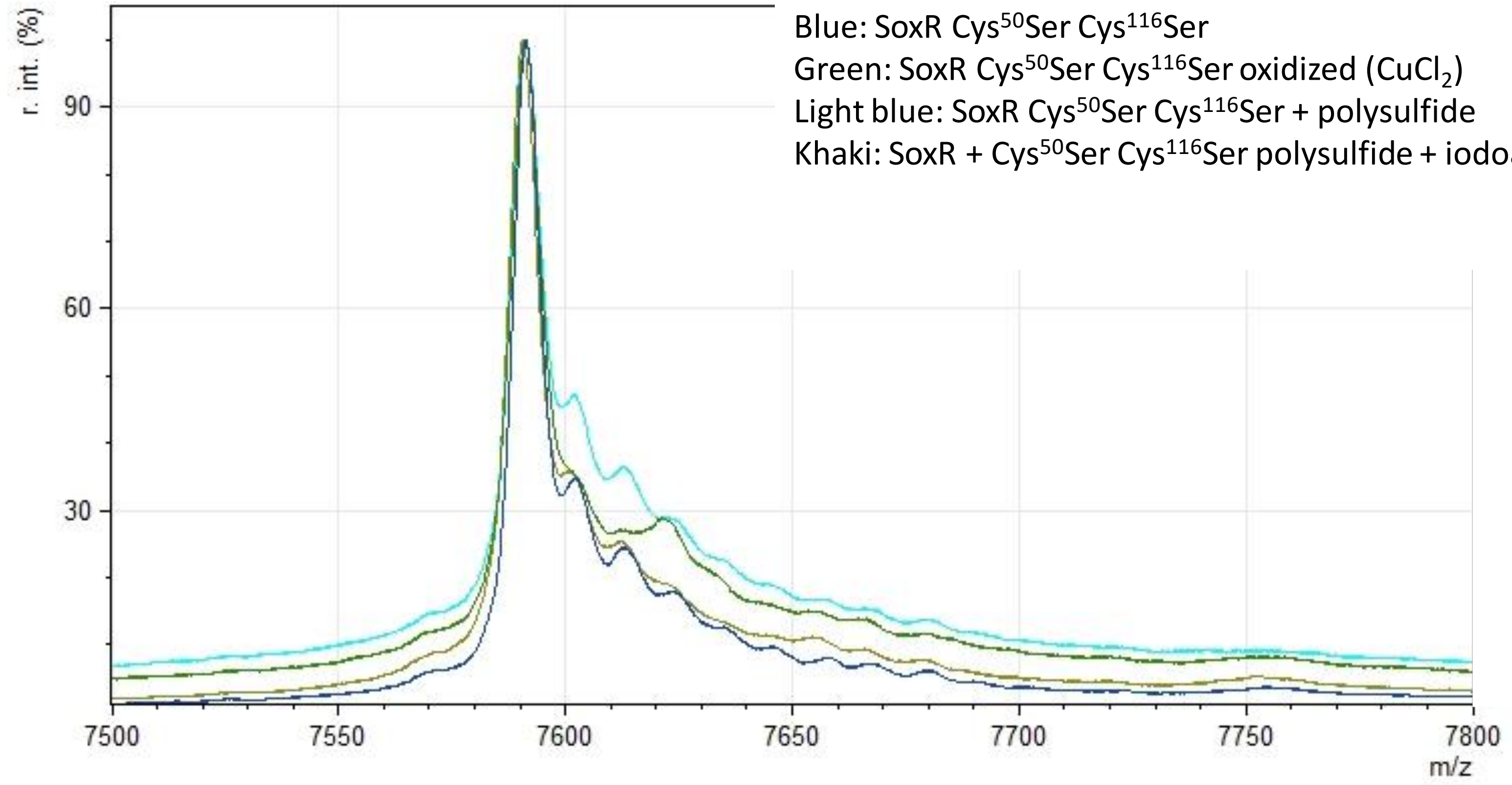

Blue: SoxR Cys^{50}Ser Cys^{116}Ser
Green: SoxR Cys^{50}Ser Cys^{116}Ser oxidized ($CuCl_2$)
Light blue: SoxR Cys^{50}Ser Cys^{116}Ser + polysulfide
Khaki: SoxR + Cys^{50}Ser Cys^{116}Ser polysulfide + iodoacetamide

Masses are shown for the *mz*/2 species.
