# Supplementary material for: In the Alphaproteobacterium Hyphomicrobium denitrificans SoxR Serves a Sulfane Sulfur-Responsive Repressor of Sulfur Oxidation
Source: Antioxidants (Basel). 2023 Aug 16;12(8):1620. doi: 10.3390/antiox12081620 (PMC10451225; doi:10.3390/antiox12081620)
Supplement: Supplementary file 1 [file antioxidants-12-01620-s001.zip › Table S1 strains, plasmids and primers.pdf]

Table S1. Strains, plasmids and primers

Strains primers or plasmids	Relevant genotype, description or sequence	Reference or source
**Strains**		
E. coli 10-beta	*Δ(ara-leu) 7697 araD139 fhuA ΔlacX74 galK16 galE15 e14- φ80*d*lacZΔM15 recA1 relA1 endA1 nupG rpsL* (Str^R) *rph spoT1 Δ(mrr-hsdRMS-mcrBC)*	New England Biolabs
E. coli DH5α	F⁻ φ80*lac*ZΔM15 Δ(*lac*ZYA-*arg*F)U169 *rec*A1 *end*A1 *hsd*R17(r_{K}^{-}, m_{K}^{+}) *pho*A *sup*E44 λ⁻*thi*-1 *gyr*A96 *rel*A1	New England Biolabs
E. coli BL21 (DE3)	F⁻*omp*T *hsd*S_B (r_{B}^{-}, m_{B}^{-}) *gal dcm* (DE3)	Novagen
Hyphomicrobium denitrificans ΔtsdA	Sm^r, in-frame deletion of *tsdA* in *H. denitrificans* Sm200	[8]
Hyphomicrobium denitrificans ΔtsdA ΔshdrR	Sm^R, in-frame deletion of *shdrR* (Hden_0682) in *H. denitrificans ΔtsdA*	[9]
Hyphomicrobium denitrificans ΔtsdA ΔsoxR	Sm^R, deletion of *soxR* (Hden_0700) in *H. denitrificans ΔtsdA*	This work
**Primers**		This work
EMSA-Fr	TTCCCGCCCCGTCTTGGTTT	[9]
EMSA_Fr2_Fr	TCAGCGCTCGCCTGGAAGTC	This work
EMSA_Fr3_Rev	TCTAAGCATCAACATATTCATATCTTTATATATTTTCG	This work
EMSA-Rev	AGGAGTTGCATCCAAAAAAGCGTG	[9]
EMSA-Hden_0703/04-fw	GGGTCACCAAATTCTGCAGGTCTC	This work
EMSA-Hden_0703/04-rev	ATCACGCCATCTCTCCCGGAA	This work
EMSA-Hden_0699/0698-fw	AATTCCACGGCTCCGCC	This work
EMSA-Hden_0699/0698-rev	TCGACAGCTTGCGGAAATCC	This work
EMSA-sHdrR-LipS1_F	TAGAGCGAGTCTTCAGC	This work
EMSA-sHdrR-LipS1_R	CGGCCCTCTGAGAAAAG	This work
EMSA-LipX-DsrE_F	GACTTCGCCGATCAATCGATC	This work
EMSA-LipX-DsrE_R	TGCCACCTCCCCGATATG	This work
rpoB-denitf	AGGACGTGTTCACCTCGATT	[42]
rpoB-denitr	CGGCTTCGTCAAGGTTCTTC	[42]
SoxT1A 0681_qPCR-Fr	CCCGAGTGATACGATTCGCA	This work
SoxT1A 0681_qPCR-Rev	CTAAAATGCCGCCGGTGATG	This work
LplA_qPCR-Fr	GGCCATGATCGATTTGCACC	This work
LplA_qPCR-Rev	CGAGATAAATTGCACCGCCG	This work
sHdrA_qPCR-Fr	CCGATCACCATTCCGTTCGA	This work
sHdrA_qPCR-Rev	CAATTGTTTCCGGGCCGATC	This work

sHdrB2_qPCR-Fr	GACGTGGCCTACTATTCCGG	This work
sHdrB2_qPCR-Rev	CCGCGACGACAGATAGGTTT	This work
LbpA2_qPCR-Fr	GGTTCCAAGAGCAGCCTGAT	This work
LbpA2_qPCR-Rev	TCGTTGATCTCCAGAACCGC	This work
SoxXA_qPCR-Fr	CGGCGCTCATTACCTATCTC	This work
SoxXA_qPCR-Rev	TCGGGGTGTCTTTTTCAGTC	This work
TusA_qPCR-Fr	TCTGACAGTTGATGCCAAGG	This work
TusA_qPCR-Rev	CGTTTCCTCATGTTCAAGCA	This work
CytP450_qPCR-Fr	CAATACGGTTCTCGGACGTT	This work
CytP450_qPCR-Rev	CATTCGTTTCCTGACGAGGT	This work
SoxT1B (0699)_qPCR-Fr	GCCGCCGTCTCAGTAAATAA	This work
SoxT1B (0699)_qPCR-Rev	AGCAGAAGACGGCAGATGAT	This work
SoxR_qPCR-Fr	TGAAGCGGACGAGGAAGTAT	This work
SoxR_qPCR-Rev	GAGACTGTGGGCTGGTTGAT	This work
sHdrR_qPCR-Fr	TTAGGAAGTCCGCATCGTCT	This work
sHdrR_qPCR-Rev	GCACTCGTTGCGCAATAATA	This work
SoxY_qPCR-Fr	GTTCAGCTTGCGGACTTTTC	This work
SoxY_qPCR-Rev	GCCAATCGTCACCTTCACTT	This work
P1 fwd up hden_0700	TATA**CTGCAG**GATCAAGGACGTGGTGGCG (PstI)	This work
P2 rev up hden_0700	CTCTCTATCGTTTGCGGCTCCATTCCTATCCCTCGGTCGC	This work
P3 fwd down hden_0700	GCGCACCGAGGGATAGGAATGGAGCCGCAAACGATAGAGAG	This work
P4 rev down hden_0700	GTAC**TCTAGA**ACGAACGCTGCCAGAAGCCC (XbaI)	This work
pET22 SoxR-Strep fw	TATA**CATATG**TGGAGCCACCCGCAGTTCGAGAAAGCTAGCTCGGGCATCTTGCCAAAC (NdeI)	This work
pET22 SoxR-Strep rev	TGCT**AAGCTT**CTATCGTTTGCGGCTCGGTT (HindIII)	This work
SoxR C(50)S_fwd	CTGATCCTCTCCCTGCTCGCTG	This work
SoxR C(50)S_rev	CAGGCGGGATTCGTGAGC	This work
SoxR C(116)S_fwd	GATAAGTTTTCCCGCGAGGAAC	This work
SoxR C(116)S_rev	GTAGATGGCGCCGATGAA	This work
**Plasmids**		
pET-22b(+)	Ap^r	Novagen
pET-22b-SoxR-N-Strep	Ap^r, NdeI/HindIII fragment of amplified SoxR in Nde/HindIII of pET	This work
pET-22b-SoxR $C^{50}S$	Ap^r, pET-22b-SoxR-N-Strep with a $Cys^{50}Ser$ exchange	This work
pET-22b-SoxR $C^{116}S$	Ap^r, pET-22b-SoxR-N-Strep with a $Cys^{116}Ser$ exchange	This work
pET-22b-SoxR $C^{50}S$ $C^{116}S$	Ap^r, pET-22b-SoxR-N-Strep with $Cys^{50}Ser$ ans $Cys^{116}Ser$ exchanges	This work

pK18*mobsacB*-Tc	Km^r, Tc^r pHP45ΩTc tetracycline cassette inserted into pk18*mobsacB* using SmaI	[9]
pK18*mobsacB*_Tc_Δ*soxR*	Km^r, Tc^r, 1.04 kb SOE PCR fragment implementing deletion of nucleotides 4 to 362 of *soxR* cloned into pk18mobsacB-Tc using PstI and XbaI restriction sites	This work

References

8. Koch, T.; Dahl, C. A novel bacterial sulfur oxidation pathway provides a new link between the cycles of organic and inorganic sulfur compounds. *ISME J.* **2018**, *12*, 2479-2491.
9. Li, J.; Koch, J.; Flegler, W.; Garcia Ruiz, L.; Hager, N.; Ballas, A.; Tanabe, T.S.; Dahl, C. A metabolic puzzle: consumption of C_1 compounds and thiosulfate in *Hyphomicrobium denitrificans* X^T. *Biochim. Biophys. Acta Bioenerget*. **2022**, *1864*, 148932.
42. Martineau, C.; Mauffrey, F.; Villemur, R. Comparative analysis of denitrifying activities of *Hyphomicrobium nitrativorans*, *Hyphomicrobium denitrificans*, and *Hyphomicrobium zavarzinii*. *Appl. Environ. Microbiol.* **2015**, *81*, 5003-5014
